# Suitable bone markers assessing bone status in patients with both coronary artery disease and diabetes

**DOI:** 10.1186/s40200-016-0259-1

**Published:** 2016-09-06

**Authors:** Zhila Maghbooli, Solaleh Emamgholipour, Arash Hossein-nezhad, Mahmood Shirzad, Sattar Gorgani Firuzjaee

**Affiliations:** 1 Endocrinology and Metabolism Research Center, Endocrinology and Metabolism Clinical Sciences Institute, Tehran University of Medical Sciences, Tehran, Iran; 2 Clinical Biochemistry Department, Faculty of Medicine, Tehran University of Medical Sciences, Tehran, Iran; 3 Osteoporosis Research Center, Endocrinology and Metabolism Clinical Sciences Institute, Tehran University of Medical Sciences, Tehran, Iran; 4 Heart Center, Tehran University of Medical Sciences, Tehran, Iran; 5 Department of Medical Laboratory Sciences, School of Allied Health Medicine, AJA University of Medical Sciences, Tehran, Iran; 6 EMRI, 5th floor, Shariati Hospital, North Karegar Avenue, P.O Box: 1411413137, Tehran, Iran

**Keywords:** Coronary artery disease, Type 2 diabetes mellitus, Bone turnover, Bone marker, OC, P1NP, CTX

## Abstract

**Background:**

We aimed to investigate the bone turnover markers in coronary artery disease (CAD) patients with and without type 2 diabetes (T2DM) in comparison with control subjects without CAD and T2DM.

**Methods:**

This cross-sectional study was performed on 45 subjects undergoing elective heart surgery; either for coronary artery bypass grafting or for valve surgery. According to angiographic results, participants were grouped in two groups with CAD (*n* = 33) and without CAD (*n* = 12).

The serum levels of osteocalcin (OC), procollagen I aminoterminal propeptide (P1NP) and carboxy-terminal collagen crosslinks (CTX), as bone turnover markers, as well as serum levels of 25 (OH) vitamin D3, PTH, and common metabolic factors were analyzed in all participants.

**Results:**

Serum levels of bone markers did not differ in patients with CAD compared to non-CAD subjects. Regarding metabolic factors, serum levels of FBG had invert correlation with OC in CAD patients (*p* = 0.004). The data of subgroup analysis showed serum levels of OC and CTX were statistically significant lower in CAD-DM than CAD-non DM (*p* < 0.05). There were not any significant differences in the P1NP levels between groups.

**Conclusions:**

Our data suggest that CTX and OC would be used as suitable bone markers in CAD patients with T2DM. However, further clinical studies need to establish the role of these markers in CAD patients with diabetes.

## Background

Type 2 diabetes patients are high susceptible to cardiovascular disease, which is attributable to hyperglycemia, oxidative stress, and inflammation [1–3]. Both disorders are interrelated to other common disorders such as osteoporosis. Cross talk among these conditions proposes shared biological mechanisms, or common risk factors too [4, 5].

Circulating bone turnover markers (BTMs) mirror the current bone remodeling phase. There is also evidence that skeleton as an endocrine system has a pivotal role in regulating energy metabolism via producing BTMs. Current researches have suggested osteocalcin (OC) as a non-collagenous protein regulates glucose, lipid and energy metabolisms as well as bone metabolism [6]. Most researches have focused on the serum levels of OC in obesity, metabolic disorder and T2DM conditions [7]. Moreover, the close relation of circulating OC and atherosclerotic plaque calcification was previously demonstrated. It has been shown that increased endothelial progenitor cells carrying OC and monocytes expressing OC are associated with plaque vulnerability in patients with early atherosclerosis [8, 9].

Procollagen type 1 N-terminal propeptide (P1NP) is also another protein which is secreted by osteoblasts during the collagen type I synthesis. This protein has been taken into account in the osteoporosis management [10]. The serum markers of collagen I and III as the N-terminal propeptides of type I and III procollagen (PINP and PIIINP) have been suggested to serve useful markers of cardiac collagen turnover [11]. An extracellular matrix predominantly comprises of type I and III collagen and surrounds cardiac myocytes. Excess myocardial collagen deposition has been linked to cardiac hypertrophy, myocardial hibernation, myocardial infarction (MI) and congestive heart failure [11]. Moreover, it has also reported that lower levels of P1NP are associated with hyperinsulinemia and hyperglycemia in healthy individuals [12].

C-terminal cross-linked telopeptide of type-I collagen (CTX) as a bone resorptive marker is produced by osteoclasts during bone resorption. There are inconsistent results about the association of CTX and diabetes. Recently a meta-analysis diabetes patients reported the serum levels of OC and CTX were lower in diabetes patients compare to controls [13]. Moreover, it has been demonstrated CTX is a predictive factor of an increased carotid intima-media thickness in the elderly population [14].

However, little is known regarding the association of BTMs with T2DM and CAD. Hence, we aimed to investigate the BTMs in CAD patients with and without T2DM in comparison with control subjects without CAD and T2DM.

## Methods

### Patient selection

The current cross-sectional study included 45 individuals who underwent an elective heart surgery; either for coronary artery bypass grafting or for valve surgery, between September 2011 and March 2012 at the Heart hospital affiliated to Tehran University of Medical Sciences. Exclusion criteria *w*ere endocrine disorders, inflammatory diseases, and history of previous heart attack, malignancies, and known chronic diseases with the exception of classic risk factors of CAD. The ethics committee of Endocrinology and Metabolism Research Institute approved the study protocol. At the time of recruitment, all participants provided written informed consent to participate in all stages of the study. Clinical information was obtained using a standardized health questionnaire, and a general practitioner performed the physical examination. Angiography was performed on patients who presented with cardiac chest pain and a positive exercise test. In all cases, where luminal stenosis was more than 50 %, the participants were considered CAD patients.

The diagnosis of type 2 diabetes mellitus (DM) was carried out and/or confirmed following the American Diabetes Association criteria, which includes a fasting blood glucose ≥126 mg/dL on two separate occasions, random (non-fasting) blood glucose ≥200 mg/dL on two separate occasions or a blood glucose >200 mg/dL at 2 h during a standard oral glucose tolerance test [15].

### Biochemical measurements

To control the effect of circadian rhythm and food intake, all samples were collected from the patients in the morning at the same time and also patients fasted for 12 h before taking peripheral venous blood. Serum levels of glucose (FBG), total cholesterol (TC), high-density lipoprotein (HDL), low-density lipoprotein (LDL), triglycerides (TG), blood urea nitrogen (BUN), creatinine, magnesium (Mg), calcium (Ca), and liver tests (ALT, AST) were measured by enzymatic colorimetric assay [Pars-Asmun kits, Iran] using an auto analyzer [Hitachi 902, Japan]. Potassium (K) and sodium (Na) were measured by using a photoelectric flame photometer [ISE].

Serum 25(OH)D was measured by radioimmunoassay (RIA) (IDS); intra- and inter-assay coefficients of variation (CV) were 5.2 and 7.5 %, respectively. Intact parathyroid hormone (PTH) was measured using electrochemilumine assay (Roche). P1NP was measured by quantitative sandwich enzyme immunoassay technique (intra- and inter-assay CV was <8 and <10 %) (Causabio). Osteocalcin (N-MID Osteocalcin, intra- and inter-assay CV was 0.9 and 1.3 %, respectively) and CTX (intra- and inter-assay CV was <1.6 and <2.2 %, respectively) were measured using electrochemiluminescence assay (Roche). The electrochemiluminescence assay for OC recognizes a large N-terminal midfragment in addition to the intact molecule.

### Statistical analysis

Data were analyzed using SPSS software, version 16. As certain data were not normally distributed, including the OC, HDL, TG, PTH and P1NP levels, log transformation were applied to correct their normality distribution. The student *t*-test and one-way ANOVA were used to compare the differences in serum levels of bone markers in CAD patients with and without DM and controls. The non-parametric test was performed for data were not normality distributed including Cr, Na and Mg levels. A Pearson correlation was used to determine correlation between bone marker levels and metabolic factors. A univariate analysis from General Linear Model (GLM) was used to control confounding variable effects including age and sex. Numerical variables are reported as the mean ± standard error or median (IQR) and categorical variables are presented as number (percentages). In all tests, the level of significance was set at two-tailed *p*-values less than 0.05 (*p* < 0.05).

## Results

### Characteristics of study populations

This study included 45 patients undergoing elective heart surgery; either for coronary artery bypass grafting or for valve surgery. The mean age was 60.82 ± 1.46 years (55 % men). All subjects were considered based on angiography result; CAD patients (*n* = 33), non-CAD (*n* = 12). Twenty four of CAD patients had angiographically confirmed 3-vessel stenosis (>50 %). The clinical and biochemical characteristics of study participants are presented in Table 1.

**Table 1:** Demographic and laboratory characteristics of the study population

Characteristics	CAD *N* = 33	Non-CAD *N* = 12	*p*-value
Age- years	63.32 ± 1.44	55.29 ± 2.06	0.01
EF-%	48.10 ± 2.06	50.68 ± 13.46	0.19
Sex (Men)- *N*(%)	22 (66)	3 (25)	0.002
BMI- Kg/m^2^	27.79 ± 1.01	27.95 ± 2.91	0.95
FBG- mg/dl	98.68 ± 4.79	96.76 ± 5.22	0.81
Log.TG	4.94 ± 0.08	4.86 ± 0.13	0.63
TC- mg/dl	160.18 ± 8.27	179.50 ± 10.43	0.09
Log.HDL-mg/dl	3.58 ± 0.05	3.66 ± 0.08	0.41
LDL- mg/dl	99.93 ± 7.48	108.46 ± 8.24	0.82
AST- U/L	20.12 ± 1.07	24.61 ± 2.86	0.16
ALT- U/L	21.63 ± 2.20	21.83 ± 2.52	0.95
25(OH)D - nmol/l	59.69 ± 2.41	57.2 ± 4.14	0.61
Log.PTH-pg/ml	3.11 ± 0.08	3.60 ± 0.24	0.08
K- mmol/l	4.50 ± 0.08	5.34 ± 0.97	0.19
Na- mmol/l	144 (4.50)	142 (5.50)	0.01
Ca- mg/dl	10.01 ± 0.59	7.74 ± 0.84	0.04
Mg- mg/dl	2 (0.20)	2.1 (0.55)	0.21
BUN- mg/dl	45.62 ± 4.44	41.68 ± 9.10	0.64
Cr - mg/dl	0.91 (0.35)	0.7 (0.39)	0.38
ALP- Unit/l	78.36 ± 7.54	66.20 ± 11.84	0.39
Log.OC- ng/ml	2.82 ± 0.08	2.84 ± 0.13	0.89
Log.P1NP- pg/ml	3.89 ± 0.19	3.93 ± 0.35	0.93
CTX-ng/ml	0.28 ± 0.02	0.40 ± 0.05	0.039
Statin user- *N*(%)	17 (51)	1 (8)	0.006
Type 2 diabetes- *N*(%)	15 (45)	-	-
Hypertension- *N*(%)	16 (48)	3 (25)	0.09

Continuous and categorical variables were described as median ± SE and *N*(%), respectively. Non-normal or skewed variables were presented as median and interquartile range (IQR)- Na, Cr, Mg. Student t.test and Mann-Whitney *U* test were applied for normal and non-normal distributed variables, respectively

*CAD* Coronary artery disease, *N* number, *BMI* body mass index, *FBG* fasting blood glucose, *TG* triglycerides, *TC* total cholesterol, *HDL* high-density lipoprotein cholesterol, *LDL* low-density lipoprotein cholesterol, *ALT* alanine amino transferase, *AST* aspartate amino transferase, *PTH* parathyroid hormone, *K* Potassium, *Ca* Calcium, *Mg* Magnesium, *Na* Sodium, *OC* Osteocalcin, *CTX* carboxy-terminal collagen crosslinks, *P1NP* procollagen I aminoterminal propeptide, *Log* Natural logarithm

There were significant differences in mean ages and sex between CAD and non-CAD subjects (*p* < 0.05). There were not significant differences in the levels of total cholesterol, triglyceride, HDL, and LDL between two groups. This might be caused by current statin therapy; around 50 % of CAD patients used statins. Among CAD patients, 15 patients had history of diabetes. Non-CAD patients (control group) had not history of diabetes. There were no patients in either group taking anti-osteoporotic drugs, vitamin K or vitamin D supplementations.

### Bone turnover markers and CAD

**Fig. 1:**
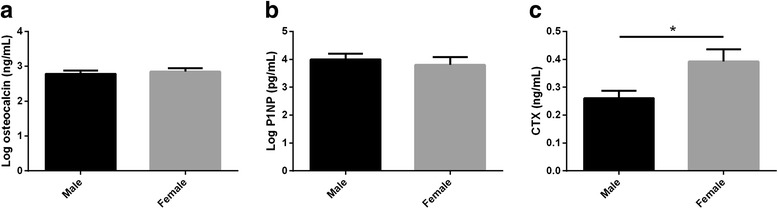
Serum levels of OC, CTX, and P1NP in male and female. Bar charts show mean ± SE levels of log-OC (**a**), log-P1NP (**b**) and CTX (**c**) in female and male. Student *t*-test was used to compare the mean serum levels of bone markers in female and male. OC; osteocalcin, CTX; cross-linked C-terminal telopeptide, P1NP; procollagen I aminoterminal propeptide, CTX; carboxy-terminal collagen crosslinks. *p-value < 0.05

Serum bone markers were evaluated in both groups; with and without CAD. No significant differences were observed in the OC and P1NP levels between CAD and non-CAD groups (*p* > 0.05). CTX levels were significantly lower in CAD patients compared to those in non-CAD patients (*p* = 0.039). To investigate the influence of sex on BTMs, all subjects were stratified based on sex. The serum levels of OC and P1NP did not significantly differ between men and women (Log OC: 2.78 ± 0.09 vs. 2.84 ± 0.10, respectively, *p* = 0.68 and Log P1NP: 3.99 ± 0.20 vs. 3.79 ± 0.28 respectively, *p* = 0.69). But, the CTX levels were lower in men than women (0.26 ± 0.02 vs. 0.39 ± 0.04, respectively, *p* = 0.01) (Fig. 1). In univariate model, after adjusting for age and sex, there was no significant association between CTX and CAD (*p* = 0.56).

### Serum bone turnover markers and metabolic factors

In all subjects, there were no significant correlation between bone markers and metabolic factors such as FBG, TC, TG, HDL and LDL. There was significant inverse correlation between OC and FBG (*r* = −0.5, *p* = 0.004) in the group of patients with CAD. There was also significant positive correlation between CTX and TG (*r* = 0.6, *p* = 0.02) in non-CAD group. In both groups examined, no correlations were found between levels of bone markers and other metabolic factors (*p* > 0.05) (Table 2). To investigate serum levels of bone markers with respect to FBG, the CAD patients were stratified according to history of T2DM; with CAD-DM (*n* = 15) and CAD-non DM (*n* = 18).

**Table 2:** Correlation between bone markers and metabolic factors

Metabolic factors	Total study population						Non-CAD group						CAD group					
	Log.OC (ng/ml)		Log.P1NP (pg/ml)		CTX (ng/ml)		Log.OC (ng/ml)		Log.P1NP (pg/ml)		CTX (ng/ml)		Log.OC (ng/ml)		Log.P1NP (pg/ml)		CTX (ng/ml)	
	r	P	r	p	r	p	R	p	R	p	R	p	r	p	r	p	r	p
FBG-mg/dl	−0.24	0.11	0.21	0.16	−0.02	0.88	0.53	0.06	−0.29	0.33	0.46	0.11	−0.50**	0.004	0.33	0.06	−0.15	0.39
TC-mg/dl	−0.15	0.39	−0.01	0.94	0.22	0.17	0.06	0.86	−0.19	0.58	0.16	0.65	−0.18	0.33	−0.02	0.88	0.22	0.22
Log.TG-mg/dl	−0.12	0.42	0.09	0.55	0.10	0.49	0.27	0.37	0.32	0.28	0.61*	0.02	−0.29	0.10	0.02	0.89	−0.12	0.50
Log.HDL-mg/dl	−0.18	0.25	0.19	0.22	−0.07	0.62	−0.49	0.08	0.18	0.55	−0.31	0.30	−0.15	0.43	0.28	0.14	−0.07	0.69
LDL-mg/dl	0.07	0.63	−0.08	0.58	0.29*	0.049	0.42	0.15	−0.27	0.37	0.41	0.16	−0.05	0.79	−0.01	0.92	0.26	0.16
BUN-mg/dl	−0.02	0.90	−0.13	0.41	−0.05	0.74	−0.18	0.71	−0.45	0.13	−0.11	0.72	0.006	0.97	0.08	0.66	−0.02	0.92
Cr-mg/dl	0.06	0.68	−0.14	0.37	−0.04	0.78	−0.12	0.68	−0.49	0.09	−0.17	0.57	0.18	0.33	0.05	0.79	0.05	0.79
BMI-kg/m^2^	0.09	0.75	−0.08	0.71	0.17	0.42	0.44	0.27	0.04	0.92	0.38	0.35	−0.43	0.11	−0.19	0.48	−0.17	0.53

*r* Pearson Correlation, *p p*-value, *CAD* Coronary artery disease, *BMI* body mass index, *FBG* fasting blood glucose, *TG* triglycerides, *TC* total cholesterol, *HDL* high-density lipoprotein cholesterol, LDL *BMD* body mass index, *Log* Natural logarithm

*Correlation is significant at the 0.05 level (2-tailed)

**Correlation is significant at the 0.01 level (2-tailed)

**Fig. 2:**
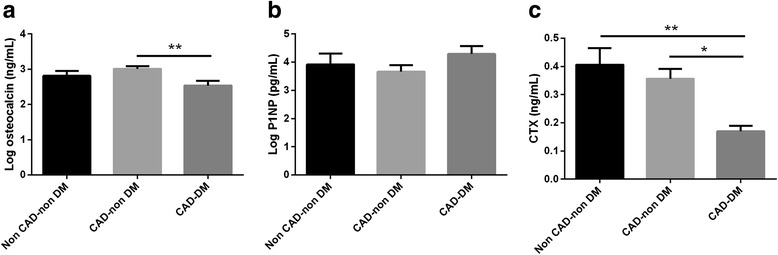
Serum levels of bone markers; in non-CAD-non DM patients, CAD-non DM and CAD-DM. Bar charts represent mean ± SE levels of log.OC (**a**), log.P1NP (**b**) and CTX (**c**) in CAD patients with and without DM, non CAD-non DM patients. One-way analysis of variance and post-hoc test were used to compare the mean serum levels of bone markers in three groups. OC; osteocalcin, CTX; cross-linked C-terminal telopeptide, P1NP; procollagen I aminoterminal propeptide, CTX; carboxy-terminal collagen crosslinks. *p-value <0.05, **p-value < 0.01

The OC levels were significantly lower in patients with both CAD and DM compared to CAD-non DM patients (Log OC: 2.52 ± 0.11 vs. 3.07 ± 0.07, respectively, *p* = 0.001). The CTX levels were also significantly lower in CAD-DM patients than those with CAD-non-DM (0.22 ± 0.03 vs. 0.35 ± 0.03, respectively, *p* = 0.019). However, in patients with both CAD and DM, we found no significant differences in mean value of serum P1NP levels compared with CAD-non DM (Log P1NP: 4.30 ± 0.28 vs. 3.58 ± 0.24, respectively, *p* = 0.19) (Fig. 2).

The mean age of CAD-DM group was similar CAD-nonDM group (63.82 ± 1.81, vs. 62.50 ± 2.12, respectively, *p* = 0.90). No significant differences in serum 25 (OH)D levels were found between two groups (58.87 ± 3.20 vs. 61.62 ± 3.37, respectively, *p* = 0.84). Also, the serum levels of PTH were not significantly differences in CAD patients with and without DM (Log PTH: 3.18 ± 0.13 vs. 3.05 ± 0.10, respectively, *p* = 0.42). Although a number of statin users was higher in CAD-DM than CAD-nonDM but it was not statistically significant (5 out of 15, vs. 12 out of 18, respectively, *p* = 0.056).

**Fig. 3:**
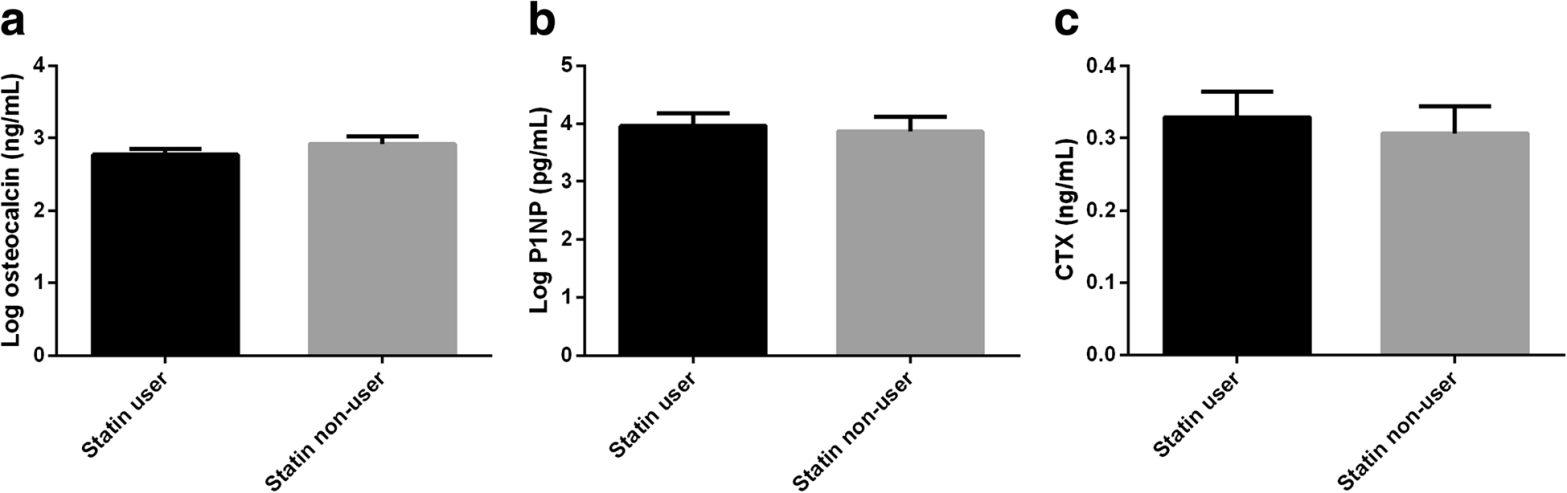
Serum levels of OC, CTX, and P1NP in statin and non-statin-users in CAD patients. Bar charts indicate mean ± SE levels of log-OC (**a**), log-P1NP (**b**) and CTX (**c**) statin and non-statin users in CAD patients. Student *t*-test was used to compare the mean serum levels of bone markers in two groups. OC; osteocalcin, CTX; cross-linked C-terminal telopeptide, P1NP; procollagen I aminoterminal propeptide, CTX; carboxy-terminal collagen crosslinks

To investigate bone marker levels with respect to lipid profiles, we stratified CAD patients based on using statins; statin-users and non-users. There were not any significant differences in the serum levels of bone markers between two groups (*p* > 0.05) (Fig. 3).

In univariate model, these statistical significances in the levels of OC and CTX were consistently observed even further adjusting for age and sex between CAD-DM and CAD-nonDM patients (*p* = 0.011, *p* = 0.013, respectively).

## Discussion

In this study we measured serum levels of OC and P1NP as bone formation markers and CTX as bone resorption marker in CAD patients and controls. Our findings showed similar levels of BTMs in CAD and non-CAD groups. There are conflicting results regarding BTMs and cardiovascular diseases. In a study by Pennisi et al., it was revealed that patients with serious atherosclerotic involvement of the carotid and/or femoral artery had decreased OC serum levels in comparison with controls, while urinary concentration of CTX and serum levels of osteoprotegerin were not significantly different between these groups [16]. Based on Poungvarin et al’s study, there was no difference in case of OC, and CTX values between CAD patients and healthy individuals [17, 18] which was compatible with our findings.

Circulating OC levels are stimulated by using anti-resorptive agents or vitamin D [19]. In our study population no patients in either group were taking anti-osteoporotic drugs, vitamin K intake or vitamin D supplementations.

Based on current studies, it has suggested that circulating levels of BTMs are changed in relation to glucose or fat metabolism [20, 21]. We found significant inverse correlation between OC and FBG in CAD patients. Moreover, it was suggested that circulating OC was inversely associated with metabolic abnormality indices including hyperglycemia, insulin resistance and carotid atherosclerosis in human studies [22–24]. Based on experimental studies hyperglycemia reduces bone turnover, in part, through impairment osteoblast function and suppression of circulating OC levels [25, 26].

Our data showed no correlation between BTMs and lipid profile in CAD patients. It might be caused by the higher percentage of current statin users in the CAD group. In subgroup analyzing by using stains, serum levels of bone markers did not significant differ between CAD statin users and non-users. A systematic review on statins and bone health in postmenopausal women showed no significant association between BTMs and using statins [27]. However, some studies have revealed using statins associate negatively with BTMs [28]. Zhi-guo and colleges’ in the study on male patients with osteopenia revealed CTX significantly reduced in atorvastatin treatment group [28].

We stratified CAD patients to DM and non-DM subgroups. As a major finding, our data showed that there was a significant difference in serum levels of CTX and OC between CAD patients with and without DM. Our findings show that both bone markers; OC and CTX decreased in patients with CAD-DM compared to CAD-non DM patients. It is suggested that changes in bone formation and resorption are not in a state of balance in CAD patients with diabetes [13].

To note, there are few studies that investigate BTMs in patients with both CAD and DM. However, there is growing evidence with conflicting results regarding evaluation of BTMs in CAD or DM and it was implicated that circulating levels of BTM are changed in these conditions.

Bao and colleges’ assessed serum levels of OC in metabolic syndrome (MS) patients [29]. MS as an aggregate of cardiometabolic risk factors is associated with increased risk of developing cardiovascular disease and diabetes [30]. They reported low serum levels of OC as a predictor factor for developing MS in Chines men. Also, serum OC was independently correlated with coronary atherosclerosis index. In another study by Ogawa-Furuya and colleges’, the serum levels of OC in patients with T2DM and abdominal aortic calcification were measured [31]. They described that the serum uncarboxilate OC levels were lower in CAD patients compared to control group ever after adjusting DM. But the levels of carboxylated OC did not differ between CAD patients and controls. In present study, we assessed N-MID osteocalcin by using electrochemilumine assay. In this method, both circulated intact and the large N-terminal/midregion fragment in serum are measured.

In term of PINP, we didn’t find any significant differences in the serum levels of P1NP in CAD patients compared to controls and also in CAD patients with and without DM. Some studies have indicated that circulating collagen metabolites are reliable surrogate in the context of atherosclerotic events, while other investigations have showed contradictory results. For example, Lin an et al., revealed the PIIINP circulating levels, but no P1NP were significantly associated with the CAD severity in patients without myocardial infraction [11]. Alla et al. reported that P1NP levels were lower in patients with hypertension and T2DM as compared to controls [32]. However, we didn’t evaluate bone markers in diabetic patients with and without CAD.

Our study has some limitations. Firstly, worked on small sample size could effect on results. Secondly, the cross-sectional design of our study limits to determine the mechanism responsible for decrease in OC in CAD-DM patients. Longitudinal studies are necessary to define the clinical relevance of bone markers in the pathogenesis of cardiovascular disease and monitoring of disease stage. Thirdly, we didn’t assess bone mineral density in study population. Evaluation of bone status should be performed to provide more evidence in this regard.

## Conclusion

In conclusion, our data showed the serum levels of OC and CTX were lower in CAD patients with T2DM. It is suggesting that OC and CTX might be as suitable bone markers to evaluate bone statues in patients with both CAD and T2DM. It is likely the hyperglycemic background in T2DM patients may lie in the plausible mechanism for decreasing OC and CTX in CAD patients.

## Abbreviations

ALP: Alkaline phosphates
BMI: Body mass index
BTMs: Bone turnover markers
BUN: Blood urea nitrogen
CAD: Coronary artery disease
CTX: C-terminal cross-linked telopeptide of type-I collagen
CV: Coefficients of variation
FBG: Fasting blood glucose
HDL: High-density lipoprotein
LDL: Low-density lipoprotein
P1NP: Procollagen I aminoterminal propeptide
RIA: Radioimmunoassay
T2DM: Type 2 diabetes mellitus
TC: Total cholesterol
TG: Triglycerides
